# Diets and leisure activities are associated with curiosity

**DOI:** 10.1371/journal.pone.0314384

**Published:** 2024-12-11

**Authors:** Satoshi Morita, Toshiaki Sueyasu, Hisanori Tokuda, Yoshihisa Kaneda, Takayuki Izumo, Kazuji Nishikawa, Takashi Kusumi, Yoshihiro Nakao

**Affiliations:** 1 Institute for Science of Life, Suntory Wellness Ltd., Kyoto, Japan; 2 Faculty of Business Administration, Department of Commerce, Osaka University of Commerce, Osaka, Japan; 3 Graduate School of Education, Kyoto University, Kyoto, Japan; University of Catania, ITALY

## Abstract

Social connections are essential for human health. While curiosity and empathy are crucial psychological factors for a fulfilling life connected with others, it is unclear if acquired environmental factors influence them. In this cross-sectional observational study, 1,311 men and women aged 20–79 years living in Japan were observed to explore how lifestyle factors such as diet, sleep, and leisure activities (such as exercise and hobbies) impact curiosity and empathy. A hierarchical multiple regression analysis revealed that diet and leisure activities impacted curiosity, whereas hobbies influenced cognitive empathy but not affective empathy. Structural equation modeling indicated that men’s curiosity was influenced by diet, leisure activities, and work, whereas women’s curiosity was influenced by leisure activities and work. These findings suggest that diet and leisure activities can enhance curiosity and cognitive empathy, leading to improved well-being.

## Introduction

Social engagement is crucial for human health [1]. Research has consistently shown that engaging with different individuals can enhance both health and overall well-being [2,3]. A lack of social relationships can severely impact health [4]. Curiosity and empathy are crucial psychological factors for attaining a rewarding, socially integrated, and prosperous life [5].

Curiosity is typically seen positively during social interactions, as it expands one’s social circle [6]. Curious individuals develop more fulfilling relationships owing to their psychological flexibility, proactivity, and self-control in social situations [6]. Moreover, curiosity can protect against negative social interactions, such as rejection, while promoting stronger long-term relationships with others [7]. Empathy plays a crucial role in socialization and forming ideal interpersonal relationships. It promotes social experiences and strengthens close bonds [8]. Empathy is a complex concept that includes emotional and cognitive components. The emotional component involves experiencing feelings for others, such as sharing others’ emotions and feeling compassion. The cognitive component involves understanding others’ thoughts and feelings [9,10]. Research has found positive relationships with curiosity, empathy, and overall health and well-being [11–15]. Understanding factors that are related to curiosity and empathy could potentially contribute to insights about leading a more fulfilling life.

However, whether curiosity and empathy are innate personality traits or acquired traits influenced by external factors and experiences remains unclear. Curiosity is a fundamental aspect of cognition, mediated by the same mechanisms as extrinsically motivated rewards [16]. In states of high curiosity, increased activity in the midbrain and nucleus accumbens has been reported. This activity pattern suggests that neural mechanisms involved in externally motivated rewards might also be involved in internally motivated curiosity [17]. Cognitive empathy is associated with neural activity in the temporoparietal junction and medial prefrontal cortex, whereas affective empathy is associated with the insula and anterior cingulate cortex [18]. Furthermore, these neural networks can be influenced by neurochemicals [18], suggesting that curiosity and empathy may be innate personality traits, but can also be influenced by external factors that activate neural networks. Therefore, identifying factors associated with curiosity and empathy, particularly those that could be acquired, might help develop effective ways to enhance these traits.

## Literature review

This literature review concentrates on lifestyle habits as acquired factors, summarizing the current understanding of their relationship with curiosity and empathy. We focus on three lifestyle habits—diet, sleep, and leisure activities—considered essential factors for a healthy and fulfilling life [19,20]. Leisure encompasses various activities, and numerous categorizations have been proposed [21–23]. Leisure activities are generally classified into two categories: physical and non-physical, with the latter further divided into social and personal activities.

### Curiosity and lifestyle factors

First, we reviewed the relationship between curiosity and diet, sleep, and leisure activities. The relationship between curiosity and diet has been examined in several studies, revealing both positive and negative associations. For instance, the effects of curiosity on unhealthy eating behaviors were investigated, suggesting that curiosity triggers motivation to seek rewards, which in turn leads to unhealthy food choices [24]. Conversely, studies have demonstrated that diets rich in vegetables and fruits can positively impact curiosity [25,26]. The intake of n-3 polyunsaturated fatty acid (PUFA) has been reported to increase exploratory behaviors [27], which are active responses to new environments or stimuli, and are reported to be closely associated with curiosity [28]. Although the precise relationship between curiosity and sleep remains unclear, evidence suggests a link between sleep and exploratory behavior [29]. A positive correlation between curiosity and physical activity has been documented at the individual level, specifically, when participants reported higher than usual levels of curiosity [13]. Despite a lack of studies directly examining the relationship between curiosity and leisure activities, curiosity is considered to promote engagement with novel and challenging situations, thereby enhancing well-being [30]. This relationship between curiosity and leisure activities could be bidirectional. Participation in various leisure activities might facilitate exposure to novel experiences and stimulate a desire for knowledge and exploration, thereby fostering curiosity. Leisure activities are not limited to mere rest and relaxation; they encompass a variety of activities that provide physical, social, and cultural stimulation, as well as arousing interest in new knowledge and experiences [23].

### Empathy and lifestyles factors

Various studies have reported on the associations between nutrients or dietary patterns and cognitive function [31], as well as the close relationship between cognitive function and empathy [32,33]. However, research demonstrating a direct link between diet and empathy is lacking. Several studies have examined the relationship between sleep and empathy. One study demonstrated that sleep deprivation decreases affective empathy [34], suggesting that sleep deprivation impairs the ability to experience empathy. Another study also suggest that sleep deprivation impairs cognitive processes, including empathy [35]. These findings highlight the importance of maintaining good sleep habits to preserve empathy. Furthermore, exercise habits are associated with empathy [36]. Interventions with exercise training have improved several components of empathy [37]. Additionally, certain non-physical leisure activities might also be associated with higher levels of empathy. For instance, in Singaporean medical students, time spent in community service and arts-related activities positively correlated with empathy [38]. In contrast, time spent on personal leisure activities, such as watching TV or movies, was associated with lower empathy. The specific content of leisure activities might be important, with cultural activities such as reading, music, and art being more beneficial for empathy than passive personal leisure activities. Collectively, these findings suggest that empathy is associated with sleep and leisure activities, whereas the association with diet remains unclear.

In summary, the review of the relationship between curiosity, empathy, and lifestyle factors has yielded several important findings despite limited studies:

- A diet rich in vegetables, fruits, and fish might positively impact curiosity. We found no studies that directly investigated the relationship between empathy and diet.
- Sleep deprivation is associated with decreased empathy. We have not found any research supporting a direct relationship between curiosity and sleep.
- Physical activity and non-physical leisure activities might enhance curiosity. Physical, social, and arts-related leisure activities might enhance empathy.

Thus, it is important to comprehensively understand how various lifestyle factors impact each component of curiosity and empathy. Curiosity and empathy are personality traits that can significantly influence an individual’s lifestyle [5]. However, we hypothesize that lifestyle factors can also impact these traits. This study aimed to investigate the associations of lifestyle factors, such as diet, sleep, and leisure activities, with empathy and curiosity. Given that empathy is a complex concept comprising both cognitive and emotional components [9], this study examined the relationship between Cognitive Empathy (Cog-E), Affective Empathy (Af-E), and lifestyle factors. Regarding types of curiosity [14,39,40], we focused on three types of curiosity, which are Diverse Curiosity (DC), Specific Curiosity (SC), and Curiosity and Exploratory (CE). DC represents intellectual curiosity towards a wide range of fields and themes. SC, on the other hand, indicates deep intellectual curiosity focused on specific fields or themes. CE represents curiosity in everyday life that extends beyond mere knowledge acquisition, encompassing a broader spectrum of exploratory behaviors and interests. These types of curiosity positively impact health and well-being [39], aiding in adaptation to complex and changing environments [41]. This study surveyed men and women aged 20–79 years living in Japan to explore the relationships between lifestyle factors, curiosity, and empathy.

## Materials and methods

### Participants

**Fig 1:**
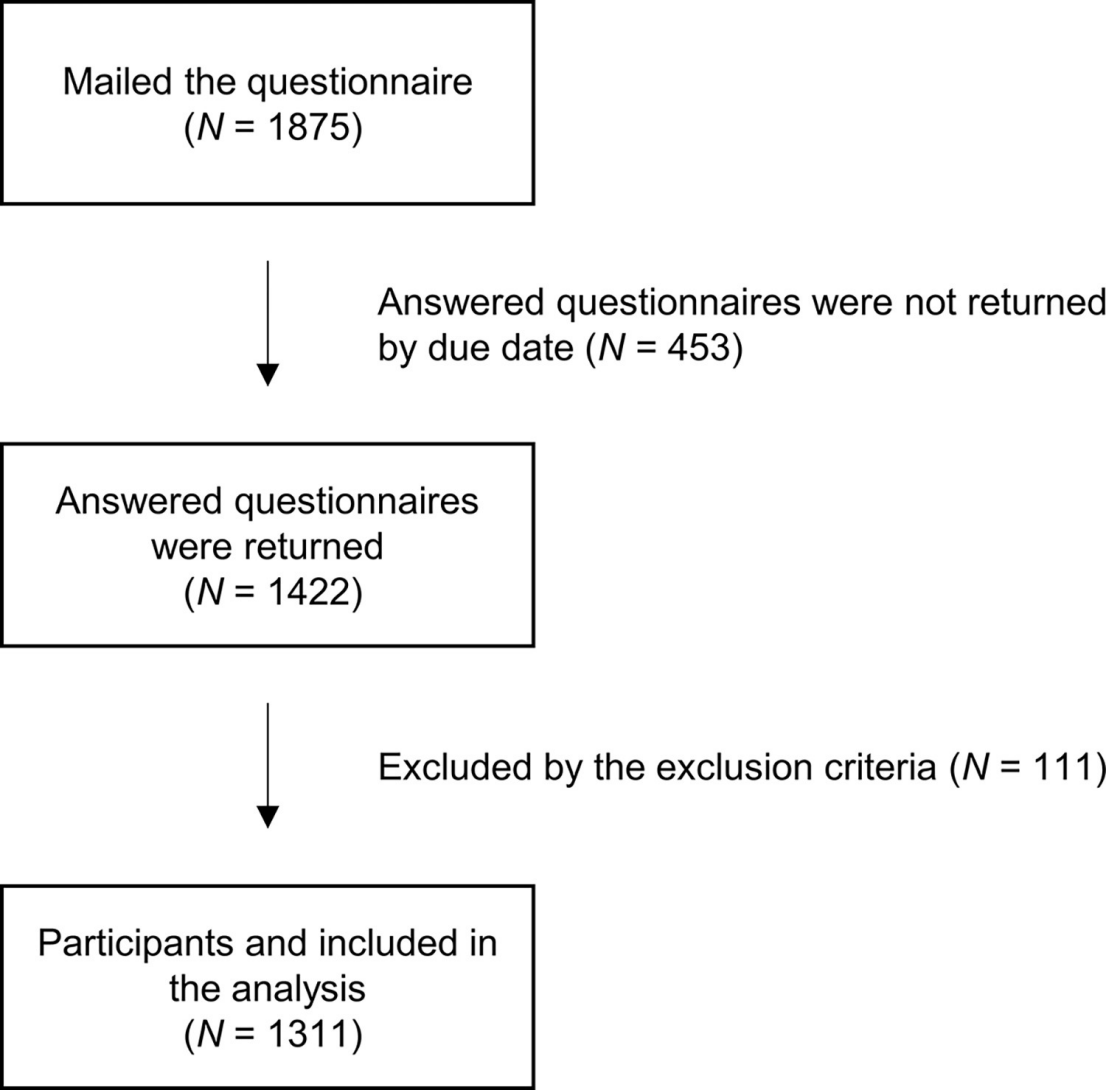
Participants included in this study. After mailing the questionnaires, we conducted the analysis on 1,311 individuals, excluding those who did not return the questionnaire by the deadline and those who met the exclusion criteria.

An observational study was conducted in Japan from February 11 to April 30, 2022. Participants were selected from individuals living in Japan who were part of a pool at Central Research Services, Inc. (Tokyo, Japan) without any specific residency restrictions. To ensure unbiased representation of age and sex, the study recruited participants in six groups: males aged 20–39, 40–59, and 60–79 years, as well as females aged 20–39, 40–59, and 60–79 years. All documents were printed and mailed. A total of 1,875 individuals received consent forms and questionnaires, and 1,422 provided signed consent and completed the questionnaires. After excluding 111 individuals based on the exclusion criteria, 1,311 participants were selected (184 males aged 20–39 years, 217 males aged 40–59 years, 215 males aged 60–79 years, 207 females aged 20–39 years, 246 females aged 40–59 years, and 242 females aged 60–79 years). Participants who returned completed questionnaires were paid a reward of 1,000 yen. A flowchart displaying the classification and breakdown of study participants is illustrated in Fig 1.

The study was conducted following the Declaration of Helsinki and was approved by the Medical Station Clinic Research Ethics Committee (protocol code 220210–1, approved on February 10, 2022). Written informed consent was obtained from all participants. The trial was registered in the University Hospital Medical Information Network (UMIN) Clinical Trial Registry (UMIN000046984). Inclusion criteria comprised women and men aged 20–79 years who understood and agreed to participate. Exclusion criteria included those diagnosed with a cranial-nerve system disease (dementia, cerebral infarction, cerebral haemorrhage, stroke, etc.) in the past, those diagnosed with a mental illness (depression, schizophrenia, anxiety disorder, etc.) in the past, those taking certain medications (sleeping pills, anxiolytics, and antidepressants), pregnant or lactating women, and those deemed inappropriate by the principal investigator as their presence could influence the outcomes of the psychological assessments [42,43].

### Psychological scales

In this study, we measured three types of curiosity and two types of empathy. The three types of curiosity assessed were Diverse Curiosity (DC), Specific Curiosity (SC), and Curiosity and Exploratory (CE). Diverse curiosity (DC) and Specific Curiosity (SC) were assessed using the Japanese version of the Epistemic Curiosity Scale [44,45]. Each item is rated on a five-point scale from “not applicable at all” (1 point) to “extremely applicable” (5 points). Mean values were calculated for DC and SC, comprising six items each. Curiosity and Exploratory (CE) were assessed using the Japanese version of the Curiosity and Exploration Inventory [46]. Each item was rated on a five-point scale, and the mean value of the nine items was then calculated. Cognitive Empathy (Cog-E) and Affective Empathy (Af-E) were measured using the Japanese version of the Interpersonal Reactivity Scale [47]. Previous research has confirmed that this scale has a four-factor structure [47]. In the present study, we focused on two of these four factors of the scale: perspective taking and empathic concern. Perspective taking is a component of Cog-E, while Empathic concern is a component of Af-E. Although the reliability of the subscales has been confirmed in previous research, it was reported that for perspective taking specifically, using 5 items instead of 7 resulted in higher reliability [47]. Consequently, we calculated perspective taking scores using these 5 items. Empathic concern was calculated using all 7 items from its subscale. The reliability and validity of the Japanese version of the Epistemic Curiosity Scale [44,45], the Japanese version of the Curiosity and Exploration Inventory [46], and the Japanese version of the Interpersonal Reactivity Scale [47] have been verified in previous research, supporting its use in the current study.

### Demographics

We included previously reported control variables or those expected to be associated with curiosity and empathy, as well as openness-related variables, a psychological trait of curiosity [48,49]. The study was conducted during the COVID-19 pandemic, and we also investigated participants’ anxiety connected to the situation. Participants provided information on their age, sex (1 = male, 2 = female), employment status (0 = not employed, 1 = employed), education level (1 = junior high school, 2 = high school, 3 = vocational school, 4 = college, 5 = graduate school), number of household members, residence area (1 = large cities and surrounding areas, 2 = local cities and surrounding areas, 3 = rural, mountain, and fishing villages), impact of the pandemic on anxiety levels (0 = no increase, 1 = increased anxiety), smoking habits (0 = non-smoker, 1 = smoker), frequency of internet use (0 = less than once a month, 1 = more than once a month), marital status (0 = unmarried, separated, or widowed, 1 = married). Alcohol consumption (g) per day was determined by utilizing questionnaires from previous administrative surveys [50]. Participants reported their daily consumption of alcohol across different types such as beer, sake, shochu, wine, whiskey, highballs, canned chu-hai, and other liquors. A free description field was included. The responses were then converted into alcohol amounts measured in grams. A positive response to any of the following four questions indicated the presence of subjective memory complaints (SMC): 1) “Do you have any difficulty with your memory?” 2) “Do you forget where you have left things more than you used to?” 3) “Do you forget the names of close friends or relatives?” and 4) “Do other people find you forgetful?” [51]. The questionnaire included household income but was excluded from the analysis as few respondents provided this information.

### Vegetables, fruits, and fish intake frequency

Based on previous studies, we focused on nutrients such as carotenoids, vitamin C, and n-3 PUFA which were considered related to curiosity [25–27]. The following questions were set up with reference to the validated brief self-administered Diet History Questionnaire (BDHQ) [52]. Participants indicated how often they consumed carotenoid-rich vegetables, vitamin C-rich fruits, and n-3 PUFA-rich fish on a seven-point scale: (1) never, (2) less than once a week, (3) once a week, (4) 2–3 times a week, (5) 4–6 times a week, (6) once a day, and (7) more than twice a day. The frequency of vegetable intake was calculated by combining the frequency of dark-green leafy vegetable consumption and carrot or pumpkin consumption over the previous month. Fruit intake frequency was determined by summing up the consumption frequencies of citrus fruits, persimmons, strawberries, kiwis, and other fruits during the preceding month. Fish intake frequency was calculated by totalling the consumption frequencies of oily fish, dried fish, salted fish, and fish paste products over the previous month. Some questions were excluded because they did not consider carotenoid-rich vegetables, vitamin C-rich fruits, or n-3 PUFA-rich fish.

### Sleep duration and sleep restfulness

The study measured sleep duration and sleep restfulness using a questionnaire previously employed in an administrative survey [50]. Participants rated their average daily sleep duration over the past month using a six-point scale (1 = less than 5 hours, 2 = 5–6 hours, 3 = 6–7 hours, 4 = 7–8 hours, 5 = 8–9 hours, 6 = more than 9 hours). Additionally, participants rated their sleep restfulness over the past month using a four-point scale (1 = never recovering from fatigue, 2 = not recovering well from fatigue, 3 = recovering from fatigue reasonably well, 4 = fully recovering from fatigue).

### Leisure activities (exercise and hobbies)

The questionnaire items for evaluating engagement in leisure activities were developed based on previous research, with modifications [23,53,54]. Exercise was defined as participation in sports, fitness, or other activities aimed at maintaining or improving health in a planned and regular manner, with each session lasting at least 30 minutes, over a period of at least one year. Commuting, shopping, and housework were excluded from the study. The listed exercises included walking, jogging, exercises (e.g., gymnastics, yoga, aerobics, tai chi), swimming, cycling, weight training, team sports (e.g., baseball, volleyball), tennis, table tennis, golf, biking, mountain climbing, and other unspecified sports activities (a free description field was available). Participants were also queried about their daily activities over the past year, excluding work and housework. Hobbies, defined as activities other than exercise, were included in the questionnaire. These activities encompassed reading books or newspapers, gardening (e.g., home gardening and Japanese bonsai gardening), playing musical instruments, choral singing, participating in creative activities (e.g., calligraphy, painting, photography, and Japanese dressmaking), art appreciation (e.g., theatre visits, watching movies, and music appreciation), writing (e.g., diary writing as a hobby), board games (e.g., shogi, trump), quizzes or puzzles (e.g., crosswords), group discussions (e.g., participating in study sessions, community gatherings, community meetings), fishing, going on trips (day or overnight), and other activities (a free description field was available). Participants reported the frequency of each exercise and hobby using the following responses: “never,” “once or several times a year,” “once or several times a month,” “once a week,” “more than twice a week,” or “more than five times a week.” Activities done more than “once or several times a year” were counted as one instance of exercise or hobby.

### Statistical analysis

To investigate the correlation between each psychological scale and the various variables, we conducted a hierarchical multiple regression analysis. Each psychological scales were treated as continuous variables. In Step 1, we included control variables such as age, sex, work, education, household members, residence area, effects of COVID-19 pandemic, alcohol intake, smoking status, marital status, internet use, and SMC. In Step 2, we added the main variables related to various lifestyles, including diets, sleep, and leisure activities. In Step 3, we included all control variables and each main variable chosen in Step 2. We hypothesized that lifestyle variables are closely linked to each psychological scale, when controlled for age, sex, work, education, household members, residence area, effects of COVID-19 pandemic, alcohol consumption, smoking, marital status, internet use, and SMC. We assessed the regression model’s goodness of fit using R-squared (*R*^*2*^), which indicates the percentage of variance in the dependent variable explained by the independent variables used in the regression model. The statistical significance of the change in *R*^*2*^ (*ΔR*^*2*^) was evaluated using an F-test. A significant F-change suggests that the variables introduced at that stage significantly enhanced the prediction [55]. The magnitude of *ΔR*^*2*^ was interpreted using the “effect size” criterion, categorized as “small” for 0.02, “moderate” for 0.13, and “large” for 0.26 [56,57]. Data were presented as mean ± standard deviation. Cronbach’s alpha was used to assess the internal consistency of each scale. Structural equation modeling (SEM) was employed to explore the relationship between different variables and lifestyle-related psychological scales. Multigroup analysis was conducted by sex. The goodness of fit of the SEM models was assessed using the comparative fit index (CFI) and the root mean square error of approximation (RMSEA). The RMSEA value in the multigroup analysis was adjusted by taking the square root of the number of groups [58]. The statistical significance level was set at *p* < .05. All analyses were conducted using SPSS Statistics version 26 and AMOS version 29 (IBM, Armonk, NY, USA).

## Results

The characteristics of the control and main variables of the study participants are presented in Tables 1 and 2. The results of the psychological scales are displayed in Table 3. Cronbach’s α was used to ensure the internal consistency of each scale. The correlation coefficients between all analysed variables can be found in S1 Table. Results for each of the six groups are included in S2–S4 Tables. The frequencies of each exercise and hobby are listed in S5 Table.

**Table 1:** Characteristics of control variables.

		Mean	(SD)
Age		51.7	(14.9)
Alcohol intake (g) per day (5 missing, 0.4%)		10.4	(21.7)
		**N**	**%**
Sex	Men	616	47.0
	Women	695	53.0
Work (12 missing, 0.9%)	Working	969	73.9
	Not working	330	25.2
Education (7 missing, 0.5%)	Junior high school graduate	20	1.5
	High school graduate	410	31.3
	Graduated from professional training college	334	25.5
	University graduate	493	37.6
	Master’s graduate	47	3.6
Household member (1 missing, 0.1%)	1	114	8.7
	2	377	28.8
	3	342	26.1
	4	296	22.6
	5	117	8.9
	6	46	3.5
	7	12	0.9
	8	6	0.5
Living area (18 missing, 1.4%)	Big cities and their suburbs	529	40.4
	Regional cities and their suburbs	731	55.8
	Farming, fishing and mountain villages	33	2.5
Effects of COVID-19 pandemic (6 missing, 0.5%)	The pandemic has increased anxiety	556	42.4
	The pandemic has not increased anxiety	749	57.1
Smoking (4 missing, 0.3%)	Smoking	190	14.5
	Not smoking	1117	85.2
Internet use (8 missing, 0.6%)	More than once a month	1069	81.5
	Less than once a month	234	17.8
Marital status (4 missing, 0.3%)	Unmarried (including separated or widowed)	358	27.3
	Married	949	72.4
Subjective memory complaints (6 missing, 0.5%)	Not SMC	576	43.9
	SMC	729	55.6

SD: Standard deviation, SMC: Subjective memory complaints

**Table 2:** Characteristics of lifestyle-related main variables.

		Mean	(SD)
Vegetable intake frequency per day (11 missing, 0.8%)		1.0	(0.7)
Fruits intake frequency per day (26 missing, 2.0%)		0.8	(0.7)
Fish intake frequency per day (7 missing, 0.5%)		0.6	(0.4)
		**N**	**%**
Number of exercise activities (9 missing, 0.7%)	0	672	51.3
	1	346	26.4
	2	192	14.6
	3	62	4.7
	4	26	2
	5	3	0.2
	6	1	0.1
Number of other hobbies (7 missing, 0.5%)	0	137	10.5
	1	256	19.5
	2	308	23.5
	3	244	18.6
	4	166	12.7
	5	96	7.3
	6	45	3.4
	7	33	2.5
	8	12	0.9
	9	6	0.5
	10	1	0.1
Sleep duration (2 missing, 0.2%)	less than 5 hours	98	7.5
	5 hours to 6 hours	396	30.2
	6 hours to 7 hours	468	35.7
	7 hours to 8 hours	283	21.6
	8 hours to 9 hours	60	4.6
	more than 9 hours	4	0.3
Sleep restfulness (2 missing, 0.2%)	Never recovering from fatigue	29	2.2
	Not recovering well from fatigue	301	23.0
	Recovering from fatigue reasonably well	647	49.4
	Fully recovering from fatigue	332	25.3

SD: Standard deviation.

**Table 3:** Psychological scales and internal consistency.

Variable		Mean	(SD)	Cronbach’s α
Diverse Curiosity (DC)	(13 missing, 1.0%)	3.16	(0.75)	.86
Specific Curiosity (SC)	(7 missing, 0.5%)	3.22	(0.75)	.86
Curiosity and Exploratory (CE)	(14 missing, 1.1%)	2.00	(0.75)	.92
Cognitive Empathy (Cog-E)	(7 missing, 0.5%)	3.28	(0.64)	.74
Affective Empathy (Af-E)	(9 missing, 0.7%)	3.71	(0.58)	.81

SD: Standard deviation.

We conducted a hierarchical multiple regression analysis to examine the key variables related to curiosity and empathy. Data from Tables 4, 5 and S6–S8 were used for DC, CE, SC, Cog-E, and Af-E, respectively. In Step 1, all control variables including age, sex, work, education, household members, residence area, the effects of the COVID-19 pandemic, alcohol consumption, smoking, internet use, marital status, and SMC were included. Subsequently, in Step 2, each main variable representing different lifestyles (vegetable intake frequency, fruit intake frequency, fish intake frequency, sleep duration, sleep restfulness, exercise, and hobby) was added. When the value of *ΔR*^*2*^ from Step 1 to Step 2 exceeded 0.02 [56,57], the main variable was selected as we deemed *ΔR*^*2*^ to be small but meaningful. DC was related to vegetable intake frequency (*ΔR*^*2*^ = .027), fish intake frequency (*ΔR*^*2*^ = .021), exercise (*ΔR*^*2*^ = .025), and hobby (*ΔR*^*2*^ = .042). Other variables were deemed unrelated to DC as *ΔR*^*2*^ from Step 1 to Step 2 was not greater than 0.02. The same analysis process was repeated, revealing relationships between CE and fish intake frequency *(ΔR*^*2*^ = .022), exercise (*ΔR*^*2*^ = .034), and hobby (*ΔR*^*2*^ = .023). SC and Cog-E were only related to the hobby (*ΔR*^*2*^ = .032 for SC, *ΔR*^*2*^ = .022 for Cog-E). Af-E was unrelated to lifestyle factors. In Step 3, all control variables and each main variable chosen in Step 2 were included for DC and CE. *ΔR*^*2*^ from Step 2 to Step 3 was greater than 0.02 (*ΔR*^*2*^ = .037 for DC, *ΔR*^*2*^ = .029 for CE).

**Table 4:** Hierarchical multiple regression analysis used to identify lifestyle factors associated with diverse curiosity (DC). Standardized coefficients.

		Diverse Curiosity (DC)								
		Step 1	Step 2							Step 3
**Control Variable**	Age	.180	.147	.135	.151	.180	.169	.143	.101	.054
	Sex	-.051	-.075	-.065	-.057	-.055	-.052	-.029	-.087	-.082
	Work	.110	.121	.128	.119	.115	.112	.121	.118	.135
	Education	.131	.112	.119	.127	.127	.126	.108	.093	.070
	Household member	.035	.023	.040	.030	.037	.036	.046	.047	.045
	Living area	-.023	-.029	-.024	-.029	-.024	-.024	-.020	-.029	-.032
	Effects of COVID-19	.021	.019	.015	.021	.023	.026	.016	.011	.007
	Alcohol intake	-.026	-.021	-.005	-.029	-.032	-.029	-.022	-.019	-.018
	Smoking	.058	.067	.076	.066	.061	.058	.071	.077	.094
	Internet use	.076	.069	.085	.079	.077	.076	.080	.054	.058
	Marriage	-.038	-.038	-.031	-.035	-.039	-.039	-.040	-.026	-.027
	SMC	-.019	-.002	-.007	-.015	-.015	-.013	-.006	-.010	.009
**Main Variable**	Vegetable intake		.170							.099
	Fruit intake			.148						
	Fish intake				.142					.076
	Sleep hours					.051				
	Sleep restfulness						.054			
	Number of exercises							.165		.120
	Number of hobbies								.222	.181
*R*		.224	.278	.265	.266	.230	.230	.274	.303	.359
*R* ^*2*^		.050	.077	.070	.071	.053	.053	.075	.092	.129
*ΔR* ^*2*^		-	**.027**	.020	**.021**	.003	.003	**.025**	**.042**	**.037**
*F*		5.415	7.834	6.984	7.180	5.262	5.275	7.597	9.501	11.119
*ΔF*		-	34.632	22.568	24.738	3.300	3.470	31.794	54.809	16.598
*p*-value of *ΔF*		-	< .01	< .01	< .01	.070	.063	< .01	< .01	< .01

DC: Diverse curiosity, SMC: Subjective memory complaints.

**Table 5:** Hierarchical multiple regression analysis used to identify lifestyle factors associated with curiosity and exploratory (CE). Standardized coefficients.

		Curiosity and Exploratory (CE)								
		Step 1	Step 2							Step 3
**Control Variable**	Age	.022	-.007	-.012	-.009	.022	.014	-.023	-.035	-.087
	Sex	-.121	-.138	-.139	-.129	-.122	-.122	-.095	-.148	-.124
	Work	.085	.095	.093	.094	.086	.087	.096	.093	.108
	Education	.113	.097	.100	.107	.112	.110	.086	.084	.064
	Household member	-.006	-.016	-.002	-.011	-.006	-.006	.006	.003	.008
	Living area	-.064	-.067	-.071	-.070	-.064	-.065	-.062	-.070	-.073
	Effects of COVID-19	-.023	-.023	-.024	-.023	-.023	-.020	-.028	-.029	-.034
	Alcohol intake	.043	.051	.042	.039	.042	.041	.047	.048	.046
	Smoking	.087	.096	.095	.096	.087	.087	.103	.103	.121
	Internet use	-.021	-.026	-.014	-.018	-.020	-.021	-.017	-.036	-.031
	Marriage	-.012	-.011	-.008	-.008	-.012	-.013	-.016	-.003	-.004
	SMC	-.002	.013	.002	-.002	-.002	.002	.013	.001	.015
**Main Variable**	Vegetable intake		.144							
	Fruit intake			.104						
	Fish intake				.149					.121
	Sleep hours					.011				
	Sleep restfulness						.036			
	Number of exercises							.193		.161
	Number of hobbies								.162	.126
*R*		.264	.296	.276	.301	.264	.266	.320	.303	.363
*R* ^*2*^		.069	.088	.076	.091	.070	.071	.103	.092	.132
*ΔR* ^*2*^		-	.019	.007	**.022**	.001	.002	**.034**	**.023**	**.029**
*F*		7.630	8.987	7.659	9.379	7.050	7.169	10.727	9.492	12.263
*ΔF*		-	25.417	11.258	27.901	.158	1.592	44.750	29.103	19.798
*p*-value of *ΔF*		-	< .01	< .01	< .01	.691	.207	< .01	< .01	< .01

CE: Curiosity and exploratory, SMC: Subjective memory complaints.

Combining multiple lifestyles may enhance associations with DC and CE, although the differences might not be substantial. Despite adding a question about COVID-19 pandemic-related anxiety to assess its impact on participants, the results did not demonstrate significant effects.

**Fig 2:**
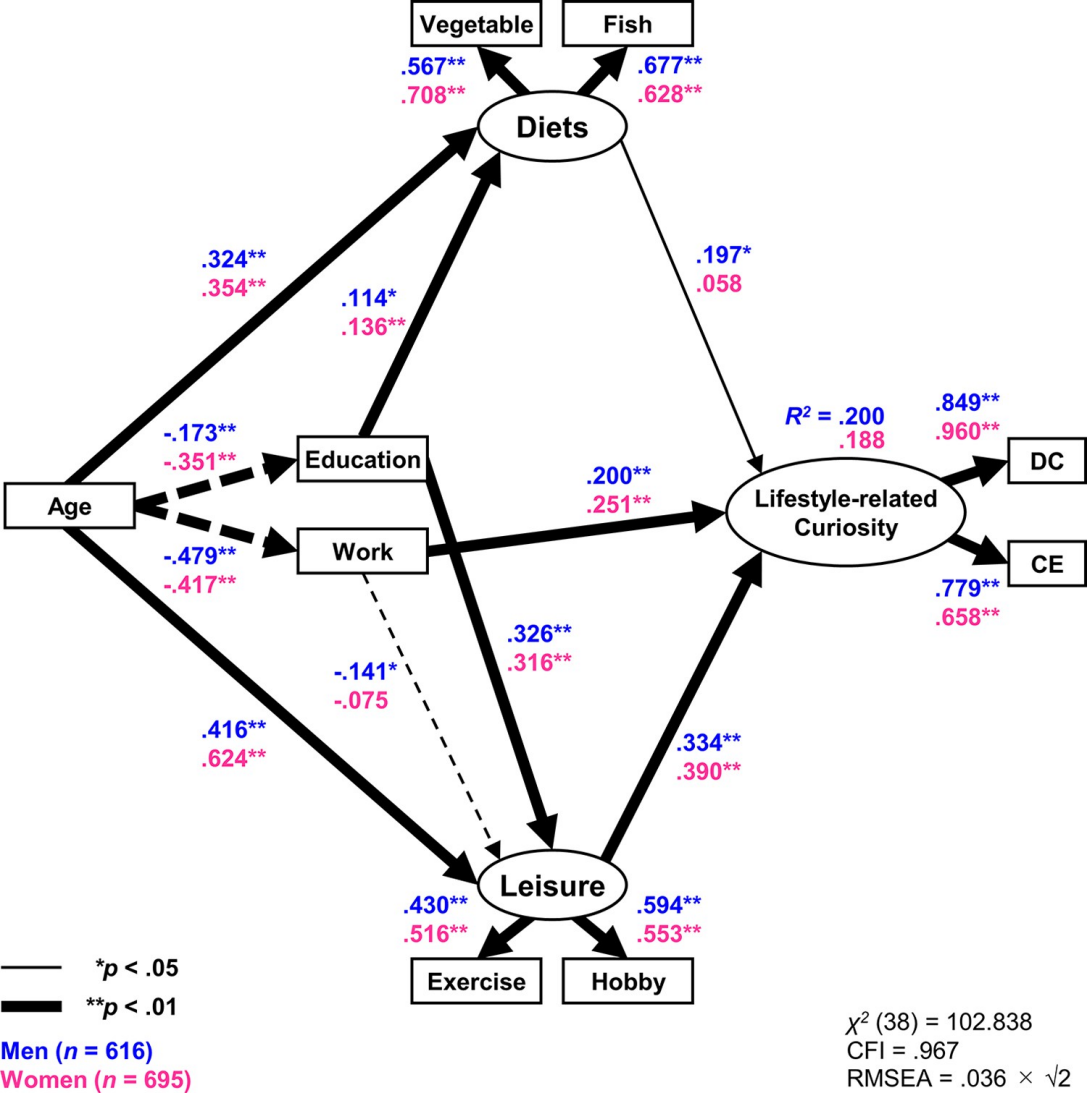
Effects of diet and leisure activities on lifestyle-related curiosity. The values on the pathway indicate the standardized path coefficients. Error variables are abbreviated in the figure. Correlations are established between error variables for diet and leisure activities. The dotted lines indicate negative effects. DC, diverse curiosity; CE, curiosity, and inventory; CFI, comparative fit index; RMSEA, root mean square error of approximation; *R*^*2*^, coefficient of determination.

Hierarchical multiple regression analysis confirmed an association between DC and CE with various lifestyle factors. To further investigate this association, we conducted an SEM analysis, as shown in Fig 2. A multigroup analysis based on gender was also performed, given the strong association of gender with the control and main variables, DC, and CE (S1–S4 Tables).

The results of the hierarchical multiple regression analysis revealed that DC and CE were particularly lifestyle-related. To account for this, we defined a latent variable called “Lifestyle-related curiosity” (L-Curiosity), with DC and CE as observed variables. Additionally, we defined the latent variable “Diet” using the frequency of vegetable and fish intake as observed variables, as these factors significantly impacted L-Curiosity. Similarly, the latent variable “Leisure activity” was defined based on the number of exercises and hobbies participated in.

Education and work were included as control variables in the model, as they demonstrated significant associations with DC, CE, diet, and leisure activities. The model’s fit was deemed acceptable with a CFI of 0.967 and an RMSEA of 0.051 (0.036×√2). The *R*^*2*^ values for L-Curiosity were 0.200 for men and 0.188 for women. In men, L-Curiosity was positively associated with diet, leisure activities, and work. In women, L-Curiosity was positively associated with leisure activities and work, while the association with diet was not significant. These results suggest that lifestyle factors, such as diet and leisure activities, impact curiosity, and significant gender differences were also observed.

## Discussion

This study conducted a cross-sectional analysis involving men and women aged 20–79 years living in Japan. Diet (vegetable and fish consumption) and leisure activities (exercise and hobbies) were positively associated with curiosity (DC and CE). Additionally, SC and Cog-E were found to be related to hobbies. However, no significant relationship was observed between Af-E and lifestyle factors. “L-Curiosity” was positively associated with work, diets, and leisure activities in men, while it was associated with work and leisure activities in women.

The results of previous reports are consistent with the present study, indicating a positive correlation between diet and curiosity [59]. Additionally, the previous observational study and an intervention study demonstrated that the consumption of vegetables and fruits led to a rise in curiosity [25,26]. Although the effect size for fruit intake was small in the current study, the result is consistent with previous research. Diets rich in antioxidants could boost curiosity by promoting the production of neurotransmitters such as dopamine, serotonin, and oxytocin [60]. In animal studies, FABP3-deficient mice show reduced novelty-seeking behaviour [61]. FABP3 transports n-3 PUFA, which is abundant in fish, suggesting a causal relationship between fish consumption and curiosity. Together, these nutrients provide a plausible explanation for our findings.

The present study did not confirm any association with sleep, whereas a previous study demonstrated a relationship between sleep deprivation and exploratory behaviour [29], and a relationship between sleep deprivation and empathy [34,35]. While previous studies have reported associations between curiosity, empathy, and sleep deprivation, in this study, individuals with short sleep duration (less than 5 hours) accounted for only 7.5% of the total participants. This might explain why our results did not coinside with those of previous research. In this study, the sleep assessment was limited to a questionnaire addressing only two points, average daily sleep duration and sleep restfulness over the past month. Objective evaluation of both sleep quantity and quality would likely allow for a more detailed analysis of the relationship with sleep. The current study discovered that engaging in leisure activities such as exercise and hobbies is associated with curiosity (DC and CE), which aligns with previous research [13,30].

Previous studies have demonstrated a positive association between exercise habits and Cog-E, but not Af-E [36]. The present study confirms that Af-E is not linked to physical exercise. No association was found between the number of exercises and Cog-E. Owing to the inability to measure physical activity in this study, we analysed the relationship with the number of exercises performed, which may have caused the divergent results.

Analysing lifestyle-related curiosity (DC and CE) using SEM suggested that combining multiple lifestyle factors, rather than focusing on a single activity, has a stronger influence on curiosity. In men, curiosity was associated with work, diet, and leisure activities. In women, it was associated with work and leisure activities. Although there are gender differences, combining multiple lifestyle factors might be more effective in increasing curiosity in both genders. The reason for gender differences in this study is unclear. Women had higher vegetable and fish intake frequencies than men. Women who already had high vegetable and fish intake frequencies may have been influenced by their leisure activities and employment status more than their diet.

The particular leisure activities most strongly associated with curiosity remain unclear. Some activities were performed by fewer people, making analysis difficult for this study population. Certain exercises and hobbies are done alone or with others either at home or outdoors. While we focused on the number of leisure activities carried out, it would be intriguing to identify the categorization of leisure activities closely associated with curiosity [21–23,62].

This study has limitations owing to its cross-sectional design, which makes it challenging to establish causal relationships between lifestyle, curiosity, and empathy. Despite this limitation, we attempted to estimate causal relationships through SEM analysis. Lifestyle may enhance certain aspects of curiosity, such as DC and CE. The study was conducted post the COVID-19 pandemic, which led to significant changes in daily life. To account for the pandemic’s impact, we included questions related to anxiety. However, it might not have adequately assessed the pandemic’s impact on anxiety levels with a dichotomous scale. As participants received printed questionnaires mailed directly to their homes, the validated dietary survey method for Japanese individuals [52] was considered challenging to accurately implement owing to the large number of questions. Thus, we used a subset of questions focusing on the frequency of consuming nutrient-rich foods associated with curiosity. Although annual income has been linked to curiosity [48], 118 respondents (9.0%) did not disclose their annual income, precluding its use it as a control variable. However, our analysis confirmed that this omission did not significantly affect the results. Interpersonal curiosity [63], defined as the motivation for exploratory behavior aimed at acquiring information about others, such as their psychological states and secrets, was not assessed in the present study. Nevertheless, considering the interpersonal aspect of curiosity would be valuable in fully understanding the relationship between empathy and curiosity. Further research would be necessary to explore the relationships among interpersonal curiosity, empathy, and lifestyle factors. Our findings indicated that DC and CE were significantly linked to lifestyle, whereas SC was only associated with hobbies. The reasons for the relatively low association of lifestyle factors on SC remain unclear. The study was conducted among Japanese residents and demonstrated that diet and leisure activities impacted curiosity. However, the generalizability of the results to other countries remains uncertain. Dietary patterns differ notably between Japan and other nations and are influenced by cultural, economic, and agricultural factors. Compared to Western countries, Japanese people consume a similar amount of vegetables, but they eat less fruit and more fish [64]. Moreover, Japanese cuisine is characterized by its emphasis on nutritional balance, often featuring small portions of various dishes in a single meal. This characterization contrasts with Western-style dining, which typically high in total energy and fat content [65]. While numerous studies have investigated the frequency and types of leisure activities in Japan [23], direct comparisons with other countries are limited. It has been noted that due to significant differences in culture and religious perspectives between Japan and other countries [23], these differences are likely reflected in leisure activities as well. These cultural differences in diet and leisure activities might profoundly impact people’s daily lifestyles, which could contribute to fostering curiosity and empathy. Therefore, further research is required to comprehend the connection between diet, leisure activities, and their impacts beyond Japan.

## Conclusion

The results demonstrate a positive correlation between diet (vegetables and fish intakes), leisure activities (exercise and hobbies), and curiosity (DC and CE). Hobbies are linked to Cog-E and SC, but not Af-E. Additionally, L-Curiosity was positively impacted by work, diet, and leisure activities in men, and by work and leisure activities in women. Therefore, diet and leisure activities may improve curiosity and cognitive empathy, ultimately benefiting overall well-being.

## Supporting information

S1 Table Correlation coefficients between all analysed variables. *p < 0.05, **p < 0.01.
(DOCX)

S2 Table Characteristics of control variables for the six groups. (DOCX)

S3 Table Lifestyle-related variables in each group. (DOCX)

S4 Table Means (SDs) of each psychological scale for the six groups. (DOCX)

S5 Table Frequency of each exercise and hobby. (DOCX)

S6 Table Hierarchical multiple regression analysis used to identify lifestyle factors associated with each psychological scale (SC). Standardized coefficients.
(DOCX)

S7 Table Hierarchical multiple regression analysis used to identify lifestyle factors associated with each psychological scale (Cog-E). Standardized coefficients.
(DOCX)

S8 Table Hierarchical multiple regression analysis used to identify lifestyle factors associated with each psychological scale (Af-E). Standardized coefficients.
(DOCX)
