# Supplementary material for: Diets and leisure activities are associated with curiosity
Source: PLoS One. 2024 Dec 11;19(12):e0314384. doi: 10.1371/journal.pone.0314384 (PMC11634007; doi:10.1371/journal.pone.0314384)
Supplement: S1 Table — *p < 0.05, **p < 0.01. (DOCX) [file pone.0314384.s001.docx]

Supporting Information

**S1 Table. Correlation coefficients between all analysed variables.** **p* < 0.05, ***p* < 0.01.

| Variables | | 1 | 2 | 3 | 4 | 5 | 6 | 7 | 8 | 9 | 10 | 11 | 12 | 13 | 14 | 15 | 16 | 17 | 18 | 19 | 20 | 21 | 22 | 23 |
| --- | --- | --- | --- | --- | --- | --- | --- | --- | --- | --- | --- | --- | --- | --- | --- | --- | --- | --- | --- | --- | --- | --- | --- | --- |
| 1 | Age | 1 |  |  |  |  |  |  |  |  |  |  |  |  |  |  |  |  |  |  |  |  |  |  |
| 2 | Sex | -.01 | 1 |  |  |  |  |  |  |  |  |  |  |  |  |  |  |  |  |  |  |  |  |  |
| 3 | Work | -.43** | -.17** | 1 |  |  |  |  |  |  |  |  |  |  |  |  |  |  |  |  |  |  |  |  |
| 4 | Education | -.25** | -.20** | .18** | 1 |  |  |  |  |  |  |  |  |  |  |  |  |  |  |  |  |  |  |  |
| 5 | Household member | -.32** | .07* | .19** | -.02 | 1 |  |  |  |  |  |  |  |  |  |  |  |  |  |  |  |  |  |  |
| 6 | Living area | .05 | -.03 | -.03 | -.15** | .00 | 1 |  |  |  |  |  |  |  |  |  |  |  |  |  |  |  |  |  |
| 7 | Effects of COVID-19 | -.03 | .02 | -.03 | .01 | .03 | .01 | 1 |  |  |  |  |  |  |  |  |  |  |  |  |  |  |  |  |
| 8 | Alcohol intake | .11** | -.28** | .10** | .01 | -.05 | -.02 | .01 | 1 |  |  |  |  |  |  |  |  |  |  |  |  |  |  |  |
| 9 | Smoking | -.01 | -.20** | .06* | -.06* | .01 | .04 | -.04 | .22** | 1 |  |  |  |  |  |  |  |  |  |  |  |  |  |  |
| 10 | Internet use | -.30** | -.06* | .20** | .19** | .08** | -.09** | .03 | .02 | -.02 | 1 |  |  |  |  |  |  |  |  |  |  |  |  |  |
| 11 | Marital status | .29** | .01 | -.11** | -.03 | .26** | -.03 | .04 | .07* | -.01 | -.01 | 1 |  |  |  |  |  |  |  |  |  |  |  |  |
| 12 | SMC | .20** | -.06* | -.05 | -.04 | -.02 | .00 | .09** | .07* | -.02 | -.04 | .15** | 1 |  |  |  |  |  |  |  |  |  |  |  |
| 13 | Vegetable intake | .16** | .16** | -.14** | .04 | .00 | .04 | .01 | -.08** | -.11** | -.03 | .08** | -.06* | 1 |  |  |  |  |  |  |  |  |  |  |
| 14 | Fruit intake | .34** | .17** | -.25** | -.03 | -.18** | .01 | .01 | -.17** | -.18** | -.15** | .03 | -.02 | .38** | 1 |  |  |  |  |  |  |  |  |  |
| 15 | Fish intake | .23** | .05 | -.15** | -.04 | -.08** | .04 | .01 | .02 | -.06* | -.09** | .05 | .03 | .42** | .36** | 1 |  |  |  |  |  |  |  |  |
| 16 | Sleep hours | .07* | .04 | -.10** | .04 | -.06* | .02 | -.03 | .08** | -.05 | -.05 | .02 | -.07* | .04 | .10** | .06* | 1 |  |  |  |  |  |  |  |
| 17 | Sleep restfulness | -.20** | .00 | .11** | -.01 | .09** | -.02 | .10** | -.05 | .00 | .06* | .06* | .09** | -.13** | -.15** | -.12** | -.49** | 1 |  |  |  |  |  |  |
| 18 | Number of exercises | .24** | -.14** | -.12** | .09** | -.17** | -.02 | .01 | .03 | -.08** | -.06* | .04 | .00 | .16** | .26** | .18** | .07* | -.14** | 1 |  |  |  |  |  |
| 19 | Number of hobbies | .30** | .14** | -.19** | .06* | -.18** | .01 | .04 | -.05 | -.13** | .02 | .02 | .02 | .25** | .28** | .19** | .07* | -.12** | .21** | 1 |  |  |  |  |
| 20 | DC | .05 | -.10** | .09** | .13** | -.02 | -.03 | .00 | .02 | .05 | .08** | -.01 | .00 | .16** | .13** | .15** | .05 | -.07* | .19** | .22** | 1 |  |  |  |
| 21 | SC | .00 | -.13** | .01 | .16** | -.06* | -.06* | .07* | -.02 | .05 | .07* | -.08** | .03 | .11** | .08** | .08** | .03 | .01 | .11** | .19** | .53** | 1 |  |  |
| 22 | CE | -.03 | -.18** | .12** | .15** | -.02 | -.06* | -.04 | .10** | .11** | .01 | -.03 | .00 | .11** | .06* | .15** | .00 | -.04 | .20** | .12** | .65** | .35** | 1 |  |
| 23 | Cog-E | .11** | .03 | -.02 | .01 | -.05 | -.04 | .05 | -.01 | .08** | -.04 | -.02 | .00 | .14** | .09** | .12** | .08** | -.10** | .11** | .18** | .33** | .32** | .32** | 1 |
| 24 | Af-E | .15** | .14** | -.07* | -.03 | -.04 | .03 | .13** | -.06* | .00 | .00 | .02 | .04 | .14** | .13** | .10** | .05 | -.05 | .06* | .20** | .23** | .21* | .15** | .45** |

DC: diverse curiosity, SC: specific curiosity, CE: curiosity and exploratory, Cog-E: cognitive empathy, Af-E: affective empathy, SMC: subjective memory complaints
