# Supplementary material for: Diets and leisure activities are associated with curiosity
Source: PLoS One. 2024 Dec 11;19(12):e0314384. doi: 10.1371/journal.pone.0314384 (PMC11634007; doi:10.1371/journal.pone.0314384)
Supplement: S2 Table — (DOCX) [file pone.0314384.s002.docx]

**S2 Table. Characteristics of control variables for the six groups.**

|  | | **20-39, men** | | **40-59, men** | | **60-79, men** | | **20-39, women** | | **40-59, women** | | **60-79, women** | |
| --- | --- | --- | --- | --- | --- | --- | --- | --- | --- | --- | --- | --- | --- |
|  |  | **Mean** | **(SD)** | **Mean** | **(SD)** | **Mean** | **(SD)** | **Mean** | **(SD)** | **Mean** | **(SD)** | **Mean** | **(SD)** |
| Age (n = 184, 217, 215, 207, 246, 242) | | 33.9 | (4.4) | 50.1 | (5.4) | 69.0 | (5.2) | 33.8 | (4.3) | 50.1 | (5.5) | 68.1 | (5.6) |
| Alcohol intake (g) per day (n = 183, 216, 214, 206, 246, 241) | | 8.9 | (18.2) | 15.7 | (26.5) | 20.1 | (25.7) | 3.7 | (11.2) | 5.2 | (13.8) | 4.0 | (11.2) |
|  | | **N** | **%** | **N** | **%** | **N** | **%** | **N** | **%** | **N** | **%** | **N** | **%** |
| Sex | Men | 184 | 100.0 | 217 | 100.0 | 215 | 100.0 | 0 | 0.0 | 0 | 0.0 | 0 | 0.0 |
|  | Women | 0 | 0.0 | 0 | 0.0 | 0 | 0.0 | 207 | 100.0 | 246 | 100.0 | 242 | 100.0 |
| Work | Working | 172 | 93.5 | 209 | 96.3 | 122 | 56.7 | 174 | 84.1 | 193 | 78.5 | 99 | 40.9 |
|  | Not in working | 8 | 4.3 | 6 | 2.8 | 93 | 43.3 | 31 | 15.0 | 51 | 20.7 | 141 | 58.3 |
|  | missing | 4 | 2.2 | 2 | 0.9 | 0 | 0.0 | 2 | 1.0 | 2 | 0.8 | 2 | 0.8 |
| Education | Junior high school graduate | 2 | 1.1 | 4 | 1.8 | 5 | 2.3 | 0 | 0.0 | 1 | 0.4 | 8 | 3.3 |
|  | High school graduate | 35 | 19.0 | 56 | 25.8 | 78 | 36.3 | 46 | 22.2 | 84 | 34.1 | 111 | 45.9 |
|  | Graduated from professional training college | 31 | 16.8 | 29 | 13.4 | 29 | 13.5 | 59 | 28.5 | 97 | 39.4 | 89 | 36.8 |
|  | University graduate | 105 | 57.1 | 108 | 49.8 | 97 | 45.1 | 93 | 44.9 | 59 | 24.0 | 31 | 12.8 |
|  | Master's graduate | 9 | 4.9 | 20 | 9.2 | 5 | 2.3 | 7 | 3.4 | 3 | 1.2 | 3 | 1.2 |
|  | missing | 2 | 1.1 | 0 | 0.0 | 1 | 0.5 | 2 | 1.0 | 2 | 0.8 | 0 | 0.0 |
| Household member | 1 | 19 | 8.8 | 25 | 11.6 | 14 | 6.8 | 8 | 3.3 | 24 | 9.9 | 19 | 8.8 |
|  | 2 | 41 | 18.9 | 108 | 50.2 | 22 | 10.6 | 48 | 19.5 | 134 | 55.4 | 41 | 18.9 |
|  | 3 | 51 | 23.5 | 62 | 28.8 | 47 | 22.7 | 73 | 29.7 | 52 | 21.5 | 51 | 23.5 |
|  | 4 | 74 | 34.1 | 12 | 5.6 | 76 | 36.7 | 74 | 30.1 | 14 | 5.8 | 74 | 34.1 |
|  | 5 | 23 | 10.6 | 3 | 1.4 | 30 | 14.5 | 31 | 12.6 | 8 | 3.3 | 23 | 10.6 |
|  | 6 | 5 | 2.3 | 2 | 0.9 | 15 | 7.2 | 9 | 3.7 | 8 | 3.3 | 5 | 2.3 |
|  | 7 | 3 | 1.4 | 2 | 0.9 | 1 | 0.5 | 2 | 0.8 | 0 | 0.0 | 3 | 1.4 |
|  | 8 | 1 | 0.5 | 1 | 0.5 | 2 | 1.0 | 1 | 0.4 | 1 | 0.4 | 1 | 0.5 |
|  | missing | 0 | 0.0 | 0 | 0.0 | 0 | 0.0 | 0 | 0.0 | 1 | 0.4 | 0 | 0.0 |
| Living area | Big cities and their suburb | 68 | 37.0 | 106 | 48.8 | 69 | 32.1 | 84 | 40.6 | 107 | 43.5 | 95 | 39.3 |
|  | Regional cities and their suburb | 110 | 59.8 | 103 | 47.5 | 135 | 62.8 | 114 | 55.1 | 135 | 54.9 | 134 | 55.4 |
|  | Farming, fishing and mountain villages | 4 | 2.2 | 4 | 1.8 | 10 | 4.7 | 4 | 1.9 | 3 | 1.2 | 8 | 3.3 |
|  | missing | 2 | 1.1 | 4 | 1.8 | 1 | 0.5 | 5 | 2.4 | 1 | 0.4 | 5 | 2.1 |
| Effects of COVID-19 pandemic | the corona disaster has increased anxiety | 75 | 40.8 | 93 | 42.9 | 88 | 40.9 | 93 | 44.9 | 108 | 43.9 | 99 | 40.9 |
|  | the corona disaster has not increased anxiety | 106 | 57.6 | 123 | 56.7 | 127 | 59.1 | 114 | 55.1 | 137 | 55.7 | 142 | 58.7 |
|  | missing | 3 | 1.6 | 1 | 0.5 | 0 | 0.0 | 0 | 0.0 | 1 | 0.4 | 1 | 0.4 |
| Smoking | Smoking | 34 | 18.5 | 63 | 29.0 | 37 | 17.2 | 15 | 7.2 | 22 | 8.9 | 19 | 7.9 |
|  | Not smoking | 148 | 80.4 | 153 | 70.5 | 178 | 82.8 | 192 | 92.8 | 224 | 91.1 | 222 | 91.7 |
|  | missing | 2 | 1.1 | 1 | 0.5 | 0 | 0.0 | 0 | 0.0 | 0 | 0.0 | 1 | 0.4 |
| Internet use | more than once for a month | 172 | 93.5 | 195 | 89.9 | 152 | 70.7 | 182 | 87.9 | 217 | 88.2 | 151 | 62.4 |
|  | less than once for a month | 12 | 6.5 | 21 | 9.7 | 62 | 28.8 | 23 | 11.1 | 27 | 11.0 | 89 | 36.8 |
|  | missing | 0 | 0.0 | 1 | 0.5 | 1 | 0.5 | 2 | 1.0 | 2 | 0.8 | 2 | 0.8 |
| Marital status | unmarried including separated or widowed | 90 | 48.9 | 47 | 21.7 | 35 | 16.3 | 96 | 46.4 | 41 | 16.7 | 49 | 20.2 |
|  | married | 93 | 50.5 | 170 | 78.3 | 180 | 83.7 | 109 | 52.7 | 205 | 83.3 | 192 | 79.3 |
|  | missing | 1 | 0.5 | 0 | 0.0 | 0 | 0.0 | 2 | 1.0 | 0 | 0.0 | 1 | 0.4 |
| Subjective memory complaints | not SMC | 108 | 58.7 | 83 | 38.2 | 61 | 28.4 | 115 | 55.6 | 117 | 47.6 | 92 | 38.0 |
|  | SMC | 75 | 40.8 | 133 | 61.3 | 153 | 71.2 | 91 | 44.0 | 129 | 52.4 | 148 | 61.2 |
|  | missing | 1.0 | 0.5 | 1.0 | 0.5 | 1.0 | 0.5 | 1.0 | 0.5 | 0.0 | 0.0 | 2.0 | 0.8 |

SD: standard deviation, SMC: subjective memory complaints
