# Supplementary material for: Diets and leisure activities are associated with curiosity
Source: PLoS One. 2024 Dec 11;19(12):e0314384. doi: 10.1371/journal.pone.0314384 (PMC11634007; doi:10.1371/journal.pone.0314384)
Supplement: S3 Table — (DOCX) [file pone.0314384.s003.docx]

**S3 Table. Lifestyle-related variables in each group.**

|  | | **20-39, men** | | **40-59, men** | | **60-79, men** | | **20-39, women** | | **40-59, women** | | **60-79, women** | |
| --- | --- | --- | --- | --- | --- | --- | --- | --- | --- | --- | --- | --- | --- |
|  |  | **Mean** | **(SD)** | **Mean** | **(SD)** | **Mean** | **(SD)** | **Mean** | **(SD)** | **Mean** | **(SD)** | **Mean** | **(SD)** |
| Vegetable intake frequency per day (n = 182, 213, 214, 206, 245, 240) | | 0.8 | (0.7) | 0.7 | (0.5) | 1.0 | (0.7) | 0.9 | (0.6) | 1.0 | (0.7) | 1.2 | (0.8) |
| Fruits intake frequency per day (n = 183, 211, 208, 202, 242, 239) | | 0.5 | (0.5) | 0.5 | (0.6) | 0.9 | (0.7) | 0.6 | (0.6) | 0.7 | (0.6) | 1.3 | (0.8) |
| Fish intake frequency per day (n = 183, 216, 215, 205, 244, 241) | | 0.5 | (0.4) | 0.5 | (0.3) | 0.7 | (0.4) | 0.5 | (0.5) | 0.5 | (0.4) | 0.7 | (0.5) |
|  | | **N** | **%** | **N** | **%** | **N** | **%** | **N** | **%** | **N** | **%** | **N** | **%** |
| Number of exercise activities | 0 | 99 | 53.8 | 102 | 47.0 | 74 | 34.4 | 167 | 80.7 | 146 | 59.3 | 84 | 34.7 |
|  | 1 | 44 | 23.9 | 58 | 26.7 | 75 | 34.9 | 24 | 11.6 | 62 | 25.2 | 83 | 34.3 |
|  | 2 | 21 | 11.4 | 40 | 18.4 | 42 | 19.5 | 13 | 6.3 | 28 | 11.4 | 48 | 19.8 |
|  | 3 | 11 | 6.0 | 12 | 5.5 | 14 | 6.5 | 3 | 1.4 | 6 | 2.4 | 16 | 6.6 |
|  | 4 | 4 | 2.2 | 3 | 1.4 | 9 | 4.2 | 0 | 0.0 | 3 | 1.2 | 7 | 2.9 |
|  | 5 | 2 | 1.1 | 0 | 0.0 | 1 | 0.5 | 0 | 0.0 | 0 | 0.0 | 0 | 0.0 |
|  | 6 | 1 | 0.5 | 0 | 0.0 | 0 | 0.0 | 0 | 0.0 | 0 | 0.0 | 0 | 0.0 |
|  | missing | 2 | 1.1 | 2 | 0.9 | 0 | 0.0 | 0 | 0.0 | 1 | 0.4 | 4 | 1.7 |
| Number of other hobbies | 0 | 32 | 17.4 | 32 | 14.7 | 8 | 3.7 | 28 | 13.5 | 28 | 11.4 | 9 | 3.7 |
|  | 1 | 52 | 28.3 | 63 | 29 | 29 | 13.5 | 39 | 18.8 | 46 | 18.7 | 27 | 11.2 |
|  | 2 | 51 | 27.7 | 53 | 24.4 | 51 | 23.7 | 61 | 29.5 | 52 | 21.1 | 40 | 16.5 |
|  | 3 | 25 | 13.6 | 35 | 16.1 | 52 | 24.2 | 35 | 16.9 | 47 | 19.1 | 50 | 20.7 |
|  | 4 | 8 | 4.3 | 18 | 8.3 | 37 | 17.2 | 27 | 13 | 35 | 14.2 | 41 | 16.9 |
|  | 5 | 5 | 2.7 | 7 | 3.2 | 24 | 11.2 | 7 | 3.4 | 18 | 7.3 | 35 | 14.5 |
|  | 6 | 6 | 3.3 | 0 | 0.0 | 8 | 3.7 | 1 | 0.5 | 8 | 3.3 | 22 | 9.1 |
|  | 7 | 2 | 1.1 | 6 | 2.8 | 3 | 1.4 | 5 | 2.4 | 6 | 2.4 | 11 | 4.5 |
|  | 8 | 1 | 0.5 | 3 | 1.4 | 1 | 0.5 | 0 | 0.0 | 1 | 0.4 | 6 | 2.5 |
|  | 9 | 0 | 0.0 | 0 | 0.0 | 2 | 0.9 | 0 | 0.0 | 3 | 1.2 | 1 | 0.4 |
|  | 10 | 0 | 0.0 | 0 | 0.0 | 0 | 0.0 | 1 | 0.5 | 0 | 0.0 | 0 | 0.0 |
|  | missing | 2 | 1.1 | 0 | 0.0 | 0 | 0.0 | 3 | 1.4 | 2 | 0.8 | 0 | 0.0 |
| Sleep duration | less than 5 hours | 21 | 11.4 | 17 | 7.8 | 13 | 6.0 | 11 | 5.3 | 25 | 10.2 | 11 | 4.5 |
|  | 5 hours to 6 hours | 57 | 31.0 | 87 | 40.1 | 46 | 21.4 | 48 | 23.2 | 79 | 32.1 | 79 | 32.6 |
|  | 6 hours to 7 hours | 71 | 38.6 | 71 | 32.7 | 78 | 36.3 | 72 | 34.8 | 85 | 34.6 | 91 | 37.6 |
|  | 7 hours to 8 hours | 29 | 15.8 | 36 | 16.6 | 61 | 28.4 | 62 | 30.0 | 46 | 18.7 | 49 | 20.2 |
|  | 8 hours to 9 hours | 5 | 2.7 | 5 | 2.3 | 16 | 7.4 | 13 | 6.3 | 9 | 3.7 | 12 | 5.0 |
|  | more than 9 hours | 0 | 0.0 | 0 | 0.0 | 1 | 0.5 | 1 | 0.5 | 2 | 0.8 | 0 | 0.0 |
|  | missing | 1 | 0.5 | 1 | 0.5 | 0 | 0.0 | 0 | 0.0 | 0 | 0.0 | 0 | 0.0 |
| Sleep restfulness | Never recovering from fatigue | 7 | 3.8 | 4 | 1.8 | 1 | 0.5 | 6 | 2.9 | 11 | 4.5 | 0 | 0.0 |
|  | Not recovering well from fatigue | 57 | 31.0 | 59 | 27.2 | 28 | 13.0 | 63 | 30.4 | 63 | 25.6 | 31 | 12.8 |
|  | Recovering from fatigue reasonably well | 84 | 45.7 | 103 | 47.5 | 114 | 53.0 | 90 | 43.5 | 118 | 48.0 | 138 | 57.0 |
|  | Fully recovering from fatigue | 35 | 19.0 | 50 | 23.0 | 72 | 33.5 | 48 | 23.2 | 54 | 22.0 | 73 | 30.2 |
|  | missing | 1 | 0.5 | 1 | 0.5 | 0 | 0.0 | 0 | 0.0 | 0 | 0.0 | 0 | 0.0 |

SD: standard deviation
