# Supplementary material for: Diets and leisure activities are associated with curiosity
Source: PLoS One. 2024 Dec 11;19(12):e0314384. doi: 10.1371/journal.pone.0314384 (PMC11634007; doi:10.1371/journal.pone.0314384)
Supplement: S4 Table — (DOCX) [file pone.0314384.s004.docx]

**S4 Table.** **Means (SDs) of each psychological scale for the six groups.**

|  | **20-39, men** | | **40-59, men** | | **60-79, men** | | **20-39, women** | | **40-59, women** | | **60-79, women** | |
| --- | --- | --- | --- | --- | --- | --- | --- | --- | --- | --- | --- | --- |
|  | **Mean** | **(SD)** | **Mean** | **(SD)** | **Mean** | **(SD)** | **Mean** | **(SD)** | **Mean** | **(SD)** | **Mean** | **(SD)** |
| Diverse Curiosity (DC) (n = 182, 217, 213, 207, 244, 235) | 3.19 | (0.81) | 3.25 | (0.81) | 3.26 | (0.81) | 3.04 | (0.78) | 3.10 | (0.72) | 3.11 | (0.70) |
| Specific Curiosity (SC) (n = 183, 217, 215, 206, 246, 237) | 3.30 | (0.78) | 3.41 | (0.78) | 3.27 | (0.78) | 3.13 | (0.71) | 3.12 | (0.77) | 3.13 | (0.71) |
| Curiosity and Exploratory (CE) (n = 182, 217, 211, 205, 246, 236) | 2.19 | (0.82) | 2.14 | (0.82) | 2.10 | (0.82) | 1.89 | (0.75) | 1.90 | (0.70) | 1.83 | (0.69) |
| Cognitive Empathy (Cog-E) (n = 183, 217, 213, 206, 245, 240) | 3.16 | (0.77) | 3.25 | (0.77) | 3.34 | (0.77) | 3.20 | (0.68) | 3.29 | (0.59) | 3.37 | (0.58) |
| Affective Empathy (Af-E) (n = 183, 217, 212, 205, 245, 240) | 3.51 | (0.68) | 3.61 | (0.68) | 3.75 | (0.68) | 3.68 | (0.62) | 3.79 | (0.56) | 3.88 | (0.50) |

SD: standard deviation
