# Supplementary material for: Diets and leisure activities are associated with curiosity
Source: PLoS One. 2024 Dec 11;19(12):e0314384. doi: 10.1371/journal.pone.0314384 (PMC11634007; doi:10.1371/journal.pone.0314384)
Supplement: S5 Table — (DOCX) [file pone.0314384.s005.docx]

**S5 Table. Frequency of each exercise and hobby**

|  | **never** | | **once or several times a year** | | **once or several times a month** | | **once a week** | | **2-4 times a week** | | **5 times or more a week** | |
| --- | --- | --- | --- | --- | --- | --- | --- | --- | --- | --- | --- | --- |
| exercises (9 missing, 0.7%) | **N** | **%** | **N** | **%** | **N** | **%** | **N** | **%** | **N** | **%** | **N** | **%** |
| walking | 929 | 70.9 | 4 | 0.3 | 23 | 1.8 | 43 | 3.3 | 139 | 10.6 | 164 | 12.5 |
| jogging | 1223 | 93.3 | 2 | 0.2 | 9 | 0.7 | 17 | 1.3 | 38 | 2.9 | 13 | 1.0 |
| exercises (e.g., gymnastics, yoga, aerobics, tai chi) | 1174 | 89.5 | 2 | 0.2 | 17 | 1.3 | 34 | 2.6 | 36 | 2.7 | 39 | 3.0 |
| swimming | 1285 | 98.0 | 7 | 0.5 | 3 | 0.2 | 2 | 0.2 | 5 | 0.4 | 0 | 0.0 |
| cycling | 1250 | 95.3 | 2 | 0.2 | 11 | 0.8 | 11 | 0.8 | 18 | 1.4 | 10 | 0.8 |
| stretch training | 1172 | 89.4 | 1 | 0.1 | 9 | 0.7 | 28 | 2.1 | 68 | 5.2 | 24 | 1.8 |
| team sports (e.g., baseball, volleyball) | 1256 | 95.8 | 7 | 0.5 | 12 | 0.9 | 16 | 1.2 | 11 | 0.8 | 0 | 0.0 |
| tennis | 1277 | 97.4 | 3 | 0.2 | 4 | 0.3 | 8 | 0.6 | 10 | 0.8 | 0 | 0.0 |
| table tennis | 1293 | 98.6 | 0 | 0.0 | 2 | 0.2 | 5 | 0.4 | 1 | 0.1 | 1.0 | 0.1 |
| golf | 1247 | 95.1 | 12 | 0.9 | 25 | 1.9 | 10 | 0.8 | 7 | 0.5 | 1 | 0.1 |
| hiking | 1239 | 94.5 | 35 | 2.7 | 20 | 1.5 | 5 | 0.4 | 3 | 0.2 | 0 | 0.0 |
| others | 1238 | 94.4 | 3 | 0.2 | 4 | 0.3 | 17 | 1.3 | 18 | 1.4 | 22 | 1.7 |
| hobbies (7 missing, 0.5%) | **N** | **%** | **N** | **%** | **N** | **%** | **N** | **%** | **N** | **%** | **N** | **%** |
| reading books | 766 | 58.4 | 46 | 3.5 | 84 | 6.4 | 59 | 4.5 | 184 | 14.0 | 165 | 12.6 |
| reading newspapers | 738 | 56.3 | 3 | 0.2 | 10 | 0.8 | 18 | 1.4 | 36 | 2.7 | 499 | 38.1 |
| gardening (e.g., home gardening and Japanese bonsai gardening) | 913 | 69.6 | 42 | 3.2 | 96 | 7.3 | 86 | 6.6 | 113 | 8.6 | 54 | 4.1 |
| playing the instruments | 1212 | 92.4 | 8 | 0.6 | 19 | 1.4 | 14 | 1.1 | 27 | 2.1 | 24 | 1.8 |
| singing in chorus | 1282 | 97.8 | 2 | 0.2 | 7 | 0.5 | 5 | 0.4 | 5 | 0.4 | 3 | 0.2 |
| creative activities (e.g., calligraphy, painting, photography, and Japanese dressmaking) | 1136 | 86.7 | 17 | 1.3 | 50 | 3.8 | 29 | 2.2 | 49 | 3.7 | 23 | 1.8 |
| art appreciation (e.g., music appreciation, theater visits, and watching movies) | 895 | 68.3 | 89 | 6.8 | 107 | 8.2 | 46 | 3.5 | 76 | 5.8 | 91 | 6.9 |
| writing (e.g., diary writing as a hobby) | 1141 | 87.0 | 7 | 0.5 | 10 | 0.8 | 18 | 1.4 | 30 | 2.3 | 98 | 7.5 |
| playing board games or card games | 1212 | 92.4 | 14 | 1.1 | 21 | 1.6 | 19 | 1.4 | 21 | 1.6 | 17 | 1.3 |
| quiz or puzzle (e.g., crossword puzzle) | 1044 | 79.6 | 18 | 1.4 | 33 | 2.5 | 57 | 4.3 | 75 | 5.7 | 77 | 5.9 |
| group discussion (e.g., participating in study groups, community meeting or conferene) | 1192 | 90.9 | 22 | 1.7 | 69 | 5.3 | 12 | 0.9 | 9 | 0.7 | 0 | 0.0 |
| going fishing | 1250 | 95.3 | 21 | 1.6 | 24 | 1.8 | 6 | 0.5 | 3 | 0.2 | 0 | 0.0 |
| going on day trips or overnight trips | 956 | 72.9 | 260 | 19.8 | 76 | 5.8 | 9 | 0.7 | 3 | 0.2 | 0 | 0.0 |
| others | 1110 | 84.7 | 6 | 0.5 | 36 | 2.7 | 28 | 2.1 | 57 | 4.3 | 67 | 5.1 |
