# Supplementary material for: Diets and leisure activities are associated with curiosity
Source: PLoS One. 2024 Dec 11;19(12):e0314384. doi: 10.1371/journal.pone.0314384 (PMC11634007; doi:10.1371/journal.pone.0314384)
Supplement: S6 Table — Standardized coefficients. (DOCX) [file pone.0314384.s006.docx]

**S6 Table. Hierarchical multiple regression analysis used to identify lifestyle factors associated with specific curiosity (SC).** Standardized coefficients

|  | | **Specific Curiosity (SC)** | | | | | | | |
| --- | --- | --- | --- | --- | --- | --- | --- | --- | --- |
|  |  | **Step 1** | **Step 2** | | | | | | |
| **Control Variable** | Age | .080 | .055 | .053 | .064 | .080 | .082 | .060 | .012 |
|  | Sex | -.112 | -.129 | -.123 | -.116 | -.114 | -.112 | -.105 | -.144 |
|  | Work | -.018 | -.011 | -.011 | -.014 | -.015 | -.018 | -.014 | -.010 |
|  | Education | .130 | .115 | .123 | .127 | .127 | .130 | .117 | .096 |
|  | Household member | -.004 | -.011 | -.002 | -.007 | -.003 | -.004 | -.002 | .008 |
|  | Living area | -.052 | -.053 | -.055 | -.056 | -.053 | -.051 | -.052 | -.058 |
|  | Effects of COVID-19 | .080 | .078 | .075 | .080 | .081 | .080 | .078 | .070 |
|  | Alcohol intake | -.080 | -.070 | -.068 | -.083 | -.084 | -.080 | -.077 | -.075 |
|  | Smoking | .066 | .071 | .075 | .070 | .067 | .065 | .070 | .083 |
|  | Internet use | .061 | .056 | .066 | .063 | .062 | .061 | .064 | .042 |
|  | Marriage | -.096 | -.095 | -.096 | -.095 | -.097 | -.096 | -.098 | -.082 |
|  | SMC | .011 | .020 | .020 | .013 | .013 | .010 | .017 | .016 |
| **Main Variable** | Vegetable intake |  | .120 |  |  |  |  |  |  |
|  | Fruit intake |  |  | .082 |  |  |  |  |  |
|  | Fish intake |  |  |  | .078 |  |  |  |  |
|  | Sleep hours |  |  |  |  | .033 |  |  |  |
|  | Sleep restfulness |  |  |  |  |  | -.008 |  |  |
|  | Number of exercises |  |  |  |  |  |  | .079 |  |
|  | Number of hobbies |  |  |  |  |  |  |  | .196 |
| *R* | | .245 | .267 | .259 | .259 | .247 | .245 | .257 | .303 |
| *R^2^* | | .06 | .071 | .067 | .067 | .061 | .060 | .066 | .092 |
| *ΔR^2^* | | - | .011 | .007 | .007 | .001 | .000 | .006 | **.032** |
| *F* | | 6.578 | 7.243 | 6.707 | 6.806 | 6.178 | 6.073 | 6.663 | 9.555 |
| *ΔF* | | - | 17.428 | 6.965 | 7.446 | 1.360 | .078 | 7.176 | 42.897 |
| *p*-value of *ΔF* | |  | < .01 | < .01 | < .01 | .244 | .78 | < .01 | < .01 |

SC: specific curiosity, SMC: subjective memory complaints
