# Supplementary material for: Diets and leisure activities are associated with curiosity
Source: PLoS One. 2024 Dec 11;19(12):e0314384. doi: 10.1371/journal.pone.0314384 (PMC11634007; doi:10.1371/journal.pone.0314384)
Supplement: S7 Table — Standardized coefficients. (DOCX) [file pone.0314384.s007.docx]

**S7 Table. Hierarchical multiple regression analysis used to identify lifestyle factors associated with cognitive empathy (Cog-E).** Standardized coefficients

|  | | **Cognitive Empathy (Cog-E)** | | | | | | | |
| --- | --- | --- | --- | --- | --- | --- | --- | --- | --- |
|  |  | **Step 1** | **Step 2** | | | | | | |
| **Control Variable** | Age | .169 | .147 | .158 | .149 | .168 | .152 | .154 | .114 |
|  | Sex | .056 | .041 | .059 | .052 | .052 | .055 | .074 | .029 |
|  | Work | .038 | .045 | .043 | .044 | .045 | .042 | .048 | .045 |
|  | Education | .054 | .041 | .053 | .051 | .047 | .047 | .045 | .025 |
|  | Household member | .008 | .000 | .011 | .005 | .011 | .010 | .018 | .019 |
|  | Living area | -.053 | -.056 | -.049 | -.056 | -.056 | -.055 | -.048 | -.059 |
|  | Effects of COVID-19 | .067 | .067 | .064 | .066 | .069 | .074 | .065 | .058 |
|  | Alcohol intake | -.038 | -.035 | -.039 | -.041 | -.048 | -.042 | -.040 | -.034 |
|  | Smoking | .111 | .118 | .117 | .116 | .116 | .112 | .121 | .125 |
|  | Internet use | -.004 | -.009 | -.006 | -.003 | -.002 | -.004 | -.004 | -.017 |
|  | Marriage | -.050 | -.051 | -.045 | -.048 | -.053 | -.053 | -.052 | -.038 |
|  | SMC | -.031 | -.019 | -.031 | -.029 | -.025 | -.021 | -.025 | -.028 |
| **Main Variable** | Vegetable intake |  | .109 |  |  |  |  |  |  |
|  | Fruit intake |  |  | .036 |  |  |  |  |  |
|  | Fish intake |  |  |  | .091 |  |  |  |  |
|  | Sleep hours |  |  |  |  | .080 |  |  |  |
|  | Sleep restfulness |  |  |  |  |  | .084 |  |  |
|  | Number of exercises |  |  |  |  |  |  | .093 |  |
|  | Number of hobbies |  |  |  |  |  |  |  | .160 |
| *R* | | .188 | .215 | .193 | .209 | .203 | .204 | .211 | .238 |
| *R^2^* | | .035 | .046 | .037 | .043 | .041 | .042 | .044 | .057 |
| *ΔR^2^* | | - | .011 | .002 | .008 | .006 | .007 | .009 | **.022** |
| *F* | | 3.744 | 4.579 | 3.590 | 4.298 | 4.083 | 4.123 | 4.383 | 5.701 |
| *ΔF* | | - | 14.009 | 1.317 | 10.011 | 7.899 | 8.398 | 9.790 | 27.757 |
| *p*-value of *ΔF* | |  | < .01 | .251 | < .01 | < .01 | < .01 | < .01 | < .01 |

Cog-E: cognitive empathy, SMC: subjective memory complaints
