# Supplementary material for: Diets and leisure activities are associated with curiosity
Source: PLoS One. 2024 Dec 11;19(12):e0314384. doi: 10.1371/journal.pone.0314384 (PMC11634007; doi:10.1371/journal.pone.0314384)
Supplement: S8 Table — Standardized coefficients. (DOCX) [file pone.0314384.s008.docx]

**S8 Table. Hierarchical multiple regression analysis used to identify lifestyle factors associated with affective empathy (Af-E).** Standardized coefficients

|  | | **Affective Empathy (Af-E)** | | | | | | | |
| --- | --- | --- | --- | --- | --- | --- | --- | --- | --- |
|  |  | **Step 1** | **Step 2** | | | | | | |
| **Control Variable** | Age | .223 | .211 | .209 | .211 | .222 | .212 | .220 | .172 |
|  | Sex | .150 | .138 | .144 | .147 | .147 | .150 | .158 | .130 |
|  | Work | .024 | .029 | .023 | .026 | .029 | .026 | .030 | .030 |
|  | Education | .038 | .029 | .034 | .036 | .032 | .033 | .033 | .020 |
|  | Household member | .046 | .041 | .050 | .045 | .049 | .048 | .054 | .050 |
|  | Living area | .026 | .023 | .028 | .023 | .023 | .024 | .028 | .026 |
|  | Effects of COVID-19 | .130 | .132 | .128 | .130 | .132 | .135 | .129 | .128 |
|  | Alcohol intake | -.058 | -.067 | -.059 | -.059 | -.066 | -.061 | -.059 | -.053 |
|  | Smoking | .040 | .048 | .045 | .044 | .044 | .041 | .044 | .048 |
|  | Internet use | .062 | .058 | .061 | .062 | .064 | .062 | .061 | .044 |
|  | Marriage | -.049 | -.050 | -.044 | -.048 | -.051 | -.051 | -.053 | -.044 |
|  | SMC | .009 | .019 | .008 | .011 | .014 | .016 | .013 | .019 |
| **Main Variable** | Vegetable intake |  | .079 |  |  |  |  |  |  |
|  | Fruit intake |  |  | .043 |  |  |  |  |  |
|  | Fish intake |  |  |  | .054 |  |  |  |  |
|  | Sleep hours |  |  |  |  | .064 |  |  |  |
|  | Sleep restfulness |  |  |  |  |  | .054 |  |  |
|  | Number of exercises |  |  |  |  |  |  | .041 |  |
|  | Number of hobbies |  |  |  |  |  |  |  | .136 |
| *R* | | .265 | .281 | .270 | .270 | .272 | .270 | .271 | .294 |
| *R^2^* | | .07 | .079 | .073 | .073 | .074 | .073 | .074 | .087 |
| *ΔR^2^* | | - | .009 | .003 | .003 | .004 | .003 | .004 | .017 |
| *F* | | 7.744 | 8.089 | 7.323 | 7.445 | 7.569 | 7.434 | 7.477 | 8.960 |
| *ΔF* | | - | 7.601 | 1.908 | 3.593 | 5.153 | 3.518 | 2.008 | 20.556 |
| *p*-value of *ΔF* | |  | < .01 | .167 | .058 | < .01 | .061 | .157 | < .01 |

Af-E: affective empathy, SMC: subjective memory complaints
